# Supplementary material for: The Alarm Pheromone and Alarm Response of the Clonal Raider Ant
Source: J Chem Ecol. 2023 Feb 10;49(1-2):1–10. doi: 10.1007/s10886-023-01407-4 (PMC9941220; doi:10.1007/s10886-023-01407-4)
Supplement: Supplementary file 1 — Supplementary file1 (DOCX 20 KB) [file 10886_2023_1407_MOESM1_ESM.docx]

**Table S1.** Statistical analysis of characterization of alarm behavior and localization of alarm pheromone. Table includes the statistical analyses from the quantification of features of the behavioral response of *O. biroi* colonies to a live alarmed ant and crushed body parts of an ant. Statistical comparisons were performed using a 2-way RM ANOVA with Šidák’s multiple comparisons test to compare individual timepoints.

| Experiment | Behavior | Source of variation  (Two-way RM ANOVA) | | | Number of arenas |
| --- | --- | --- | --- | --- | --- |
|  |  | Time x Stimulus | Time | Stimulus |  |
| Characterizing alarm behavior (Fig 1a-c) | Outside nest pile | 19.48%  p < 0.0001 | 24.35%  p < 0.0001 | 12.87%  p = 0.0009 | Alarmed ant  n = 13  Control paper  n = 10 |
|  | Left nest chamber | 13.71%  p < 0.0001 | 24.90%  p < 0.0001 | 6.196%  p = 0.0280 |  |
|  | Touching wall | 2.991%  p = 0.2342 | 1.872%  p = 0.5310 | 3.845%  p = 0.1928 |  |
| Localization of alarm pheromone (Fig 1d-f) | Outside nest pile | 11.75%  p < 0.0001 | 15.22%  p < 0.0001 | 29.19%  p = 0.0004 | Crushed head  n = 11  Crushed body  n = 11 |
|  | Left nest chamber | 8.380%  p = 0.0001 | 8.013%  p = 0.002 | 10.40%  p = 0.0329 |  |
|  | Touching wall | 9.666%  p < 0.0001 | 13.96%  p < 0.0001 | 31.18%  p < 0.0001 |  |

**Table S2.** Statistical analysis of EAG recordings. Antennal sensitivity to 1 μg, 10 μg, and 100 μg of 4-methyl-3-heptanone and 4-methyl-3-heptanol and a solvent control were compared using a mixed-effects analysis with a Geisser-Greenhouse correction and Dunnett’s multiple comparisons test was used to compare each compound to the solvent.

| Experiment | Mixed-effects analysis | Compound  (compared to solvent) | Adjusted P Value |
| --- | --- | --- | --- |
| Antennal sensitivity to candidate compounds  (Fig S5) | Difference between treatments  p=0.0022 | 1 μg 4-methyl-3-heptanone | p = 0.9998 |
|  |  | 10 μg 4-methyl-3-heptanone | p = 0.0451 |
|  |  | 100 μg 4-methyl-3-heptanone | p = 0.0112 |
|  |  | 1 μg 4-methyl-3-heptanol | p = 0.9085 |
|  |  | 10 μg 4-methyl-3-heptanol | p = 0.0713 |
|  |  | 100 μg 4-methyl-3-heptanol | p = 0.0100 |

**Table S3.** Statistical analysis of behavioral responses to candidate alarm pheromone components. Quantification of features of the behavioral response of *O. biroi* colonies to 4-methyl-3-heptanone, 4-methyl-3-heptanol, and a blend of 90% 4-methyl-3-heptanone and 10% 4-methyl-3-heptanol. Statistical comparisons were performed using a 2-way RM ANOVA with Dunnett’s multiple comparisons test to compare individual timepoints to the vehicle control.

| Experiment | Behavior | Source of variation  (Two-way RM ANOVA) | | | Number of arenas |
| --- | --- | --- | --- | --- | --- |
|  |  | Time x Stimulus | Time | Stimulus |  |
| Response to 4-methyl-3-heptanone  (Fig 3a-c) | Outside nest pile | 15.37%  p < 0.0001 | 37.71%  p < 0.0001 | 24.87%  p < 0.0001 | 2600 μg n = 17  260 μg n = 12  control n = 15 |
|  | Left nest chamber | 23.88%  p < 0.0001 | 23.06%  p < 0.0001 | 22.13%  p < 0.0001 |  |
|  | Touching wall | 10.59%  p < 0.0001 | 16.80%  p < 0.0001 | 6.696%  p = 0.0089 |  |
| Response to 4-methyl-3-heptanol  (Fig 3d-f) | Outside nest pile | 11.29%  p < 0.0001 | 40.97%  p < 0.0001 | 24.62%  p < 0.0001 | 2600 μg n = 11  260 μg n = 10  control n = 9 |
|  | Left nest chamber | 9.072%  p < 0.0001 | 16.99%  p < 0.0001 | 8.514%  p = 0.0510 |  |
|  | Touching wall | 4.923%  p = 0.0537 | 22.44%  p < 0.0001 | 9.693%  p = 0.0103 |  |
| Response to blend  (Fig 3g-i) | Outside nest pile | 13.11%  p < 0.0001 | 31.13%  p < 0.0001 | 34.56%  p < 0.0001 | 2600 μg n = 9  260 μg n = 8  control n = 9 |
|  | Left nest chamber | 22.31%  p < 0.0001 | 16.44%  p < 0.0001 | 24.55%  p < 0.0001 |  |
|  | Touching wall | 9.727%  p = 0.0006 | 17.46%  p < 0.0001 | 14.72%  p = 0.0037 |  |

**Table S4.** Statistical analysis of area under the curve 2 minutes following exposure to candidate alarm pheromone components and the blend. Comparison of 4-methyl-3-heptanone, 4-methyl-3-heptanol, and 90% 4-methyl-3-heptanone / 10% 4-methyl-3-heptanol blend in ants outside the nest pile, ants repelled from the compound(s), and ants attracted to the compound(s). Statistical comparisons were performed using a 2-way ANOVA with Tukey’s multiple comparisons tests to compare the different compounds and blend across concentrations.

| Behavior | Source of variation  (Two-way ANOVA) | | |
| --- | --- | --- | --- |
|  | Concentration x Compound | Concentration | Compound |
| Ants outside the nest pile – unsettled  (Fig S4a) | 0.6611%  p = 0.7941 | 57.52%  p < 0.0001 | 2.072%  p = 0.0775 |
| Ants that left the nest chamber – repulsion  (Fig S4b) | 5.335%  p = 0.1084 | 26.49%  p < 0.0001 | 0.3398%  p = 0.7803 |
| Ants that are touching the wall – attraction  (Fig S4c) | 6.088%  p = 0.0967 | 11.58%  p = 0.0008 | 14.41%  p = 0.0002 |
