# Supplementary figures and images for: The Alarm Pheromone and Alarm Response of the Clonal Raider Ant
Source: J Chem Ecol. 2023 Feb 10;49(1-2):1–10. doi: 10.1007/s10886-023-01407-4 (PMC9941220; doi:10.1007/s10886-023-01407-4)

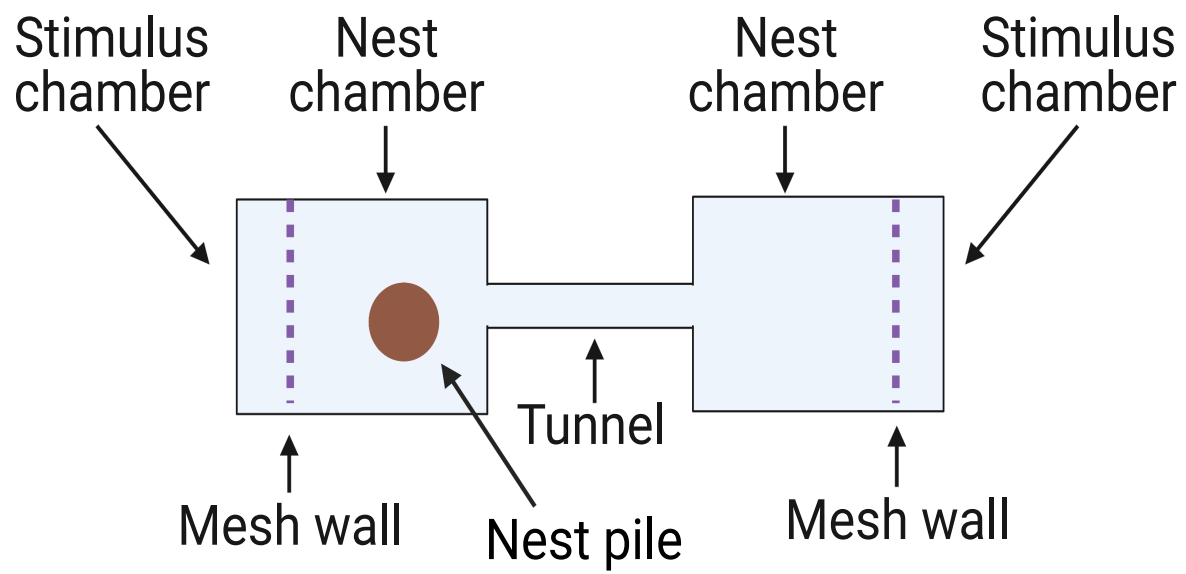

Supplement: Supplementary file 7 — Supplementary file7 (PDF 173 KB) Figure S1. Alarm arena design. The alarm arena had two areas separated by a tunnel. Each area consists of a small rectangular stimulus chamber and a large square nest chamber, separated by a mesh wall (denoted by a purple dashed line in the figure). These chambers have separate clear plastic acrylic lids, allowing access to the stimulus chamber without disturbing ants in the nest chamber. The brown circle represents the nest pile, where ants and their eggs are tightly clustered prior to starting the experiment. Created with BioRender.com [file 10886_2023_1407_MOESM7_ESM.pdf]

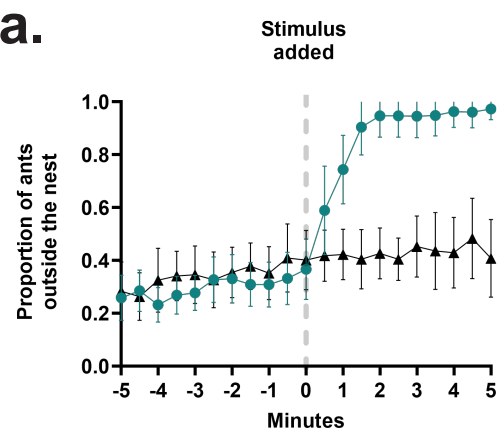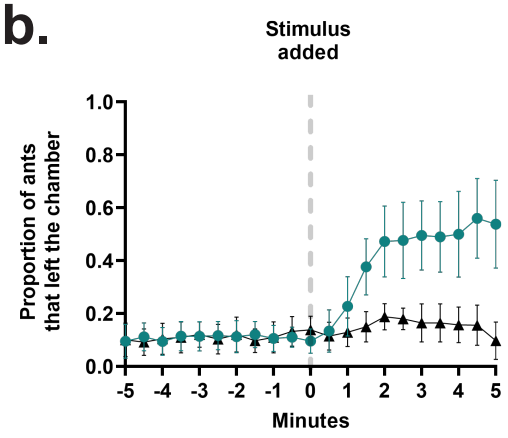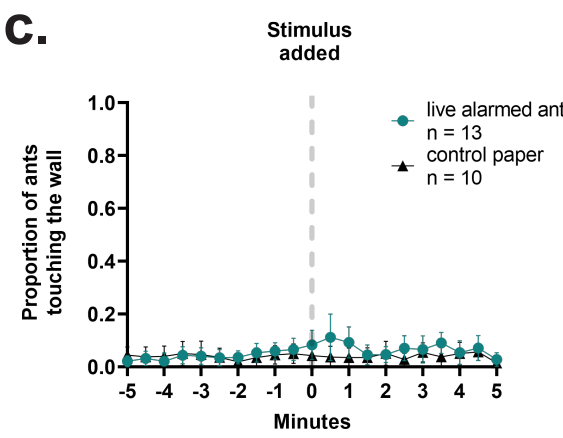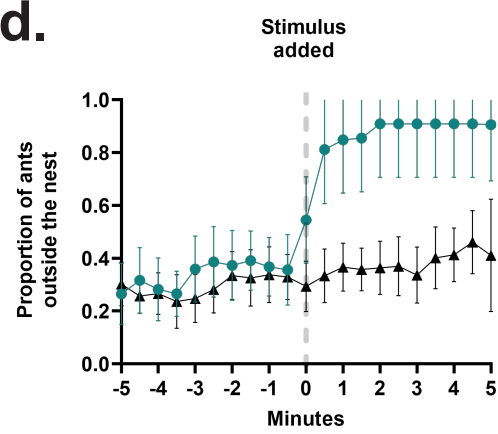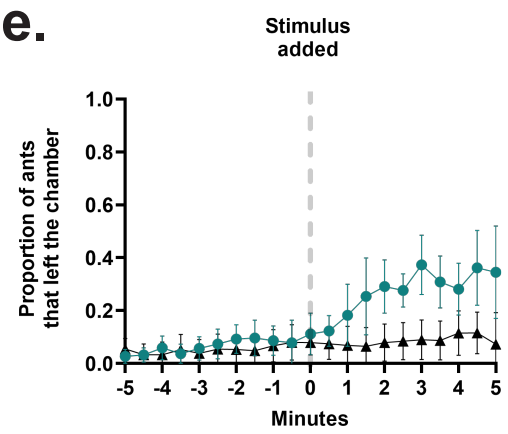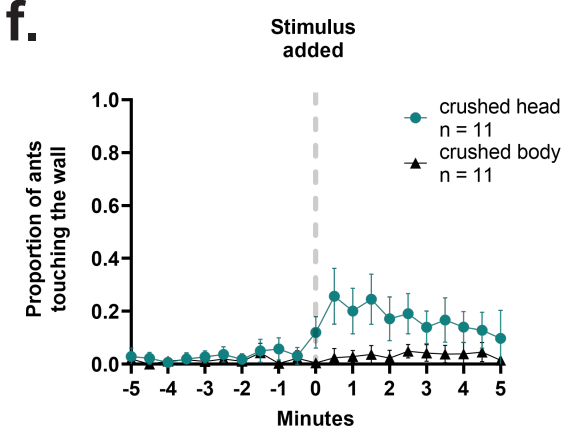

Supplement: Supplementary file 8 — Supplementary file8 (PDF 729 KB) Figure S2. Full time course of characterization of alarm behavior and localization of alarm pheromone in O. biroi. Quantification of features of the behavioral response of O. biroi colonies to a live alarmed ant (a-c) and crushed body parts of an ant (d-f). Each datapoint indicates the mean and error bars indicate the 95% confidence interval. Sample sizes represent replicate colonies tested [file 10886_2023_1407_MOESM8_ESM.pdf]

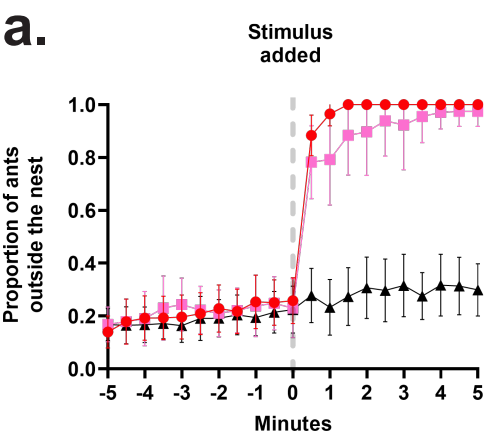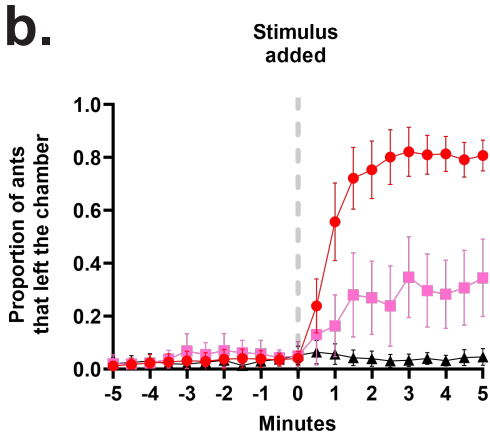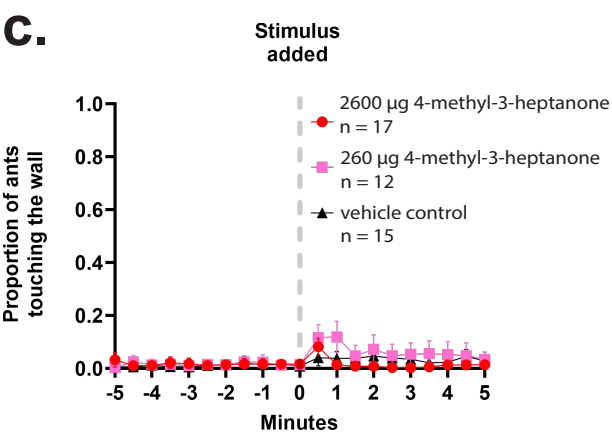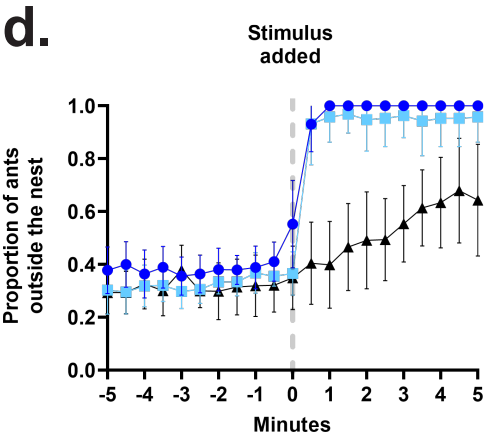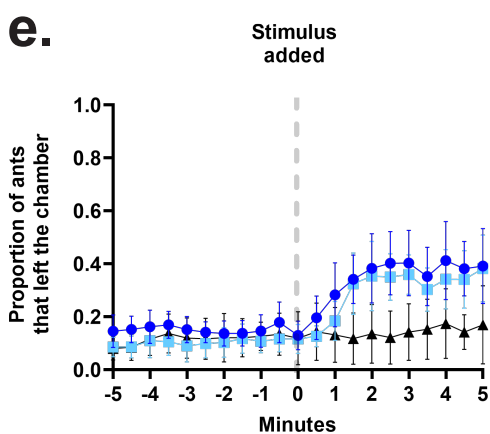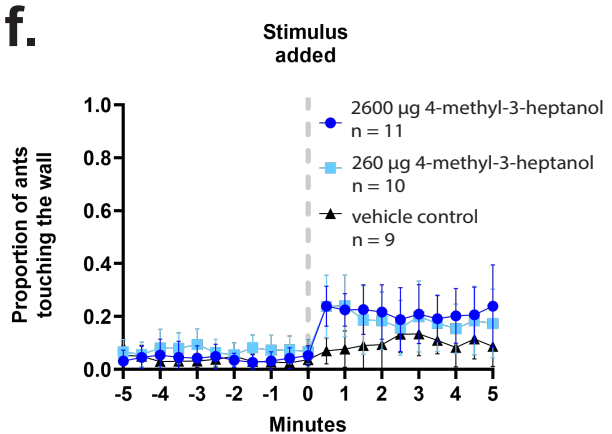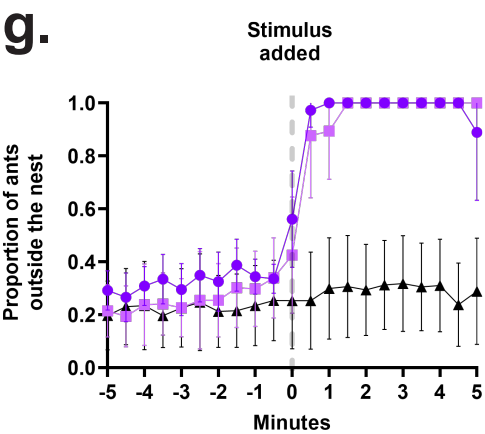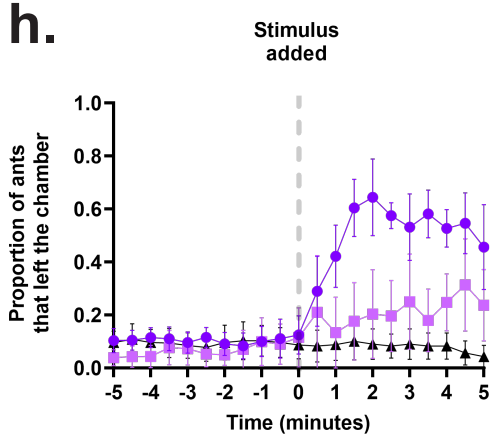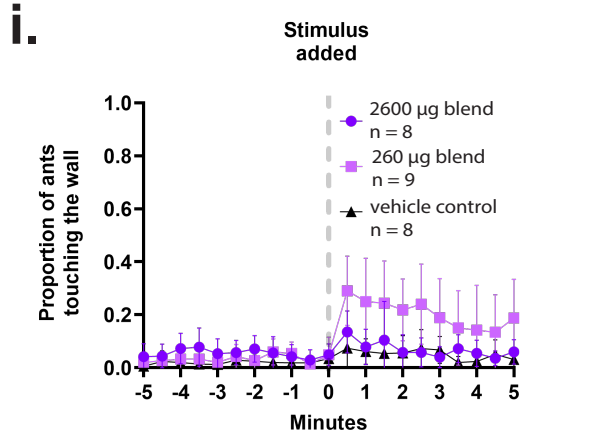

Supplement: Supplementary file 9 — Supplementary file9 (PDF 1268 KB) Figure S3. Full time course of behavioral response to candidate alarm pheromone components. Quantification of features of the behavioral response of O. biroi colonies to 4-methyl-3-heptanone (a-c), 4-methyl-3-heptanol (d-f), and a blend of 90% 4-methyl-3-heptanone and 10% 4-methyl-3-heptanol (g-i). Each datapoint indicates the mean and error bars indicate the 95% confidence interval. Sample sizes represent replicate colonies tested [file 10886_2023_1407_MOESM9_ESM.pdf]

a.

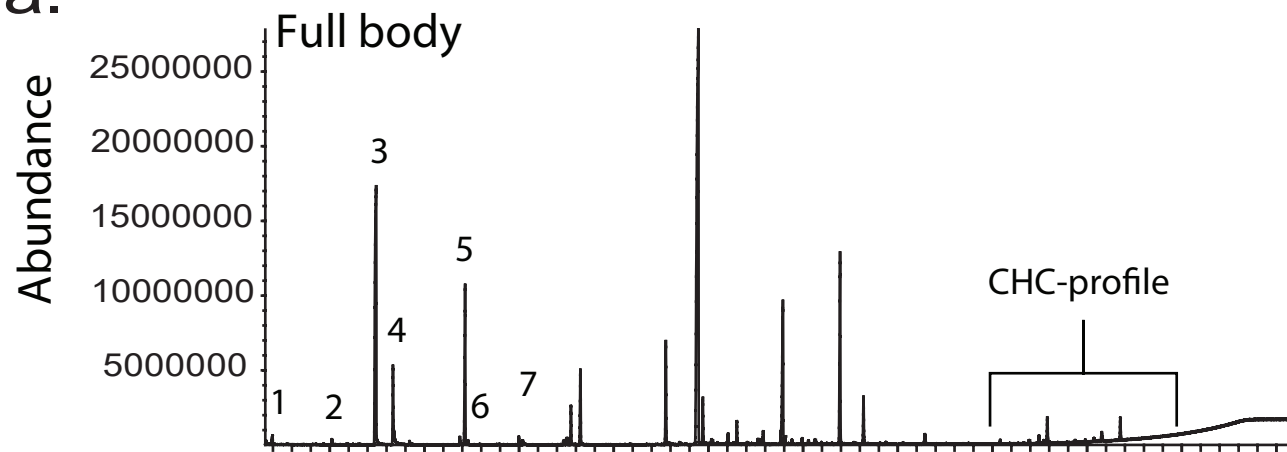

b.

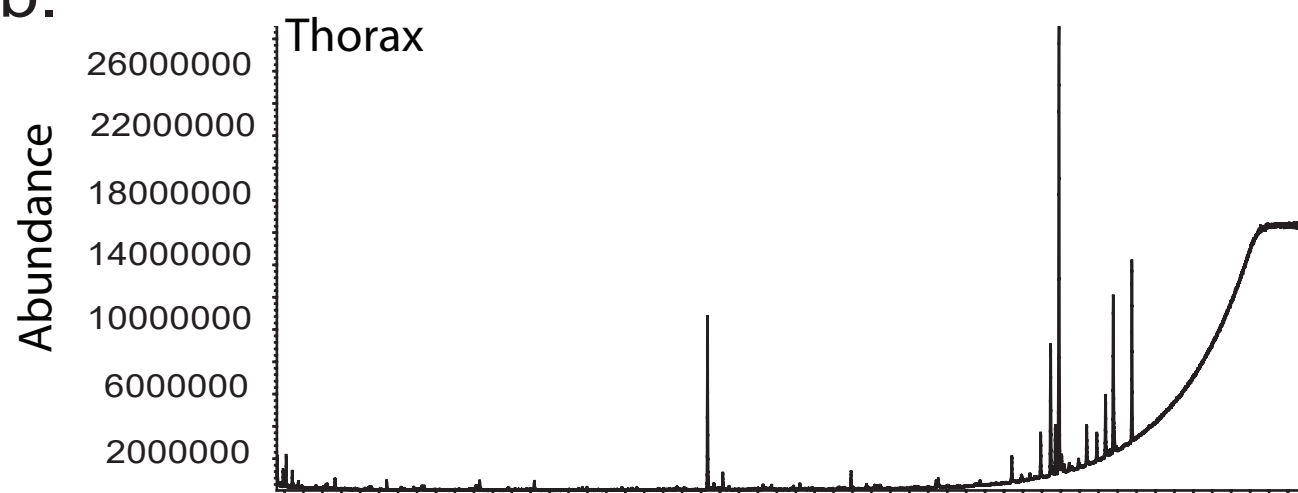

c.

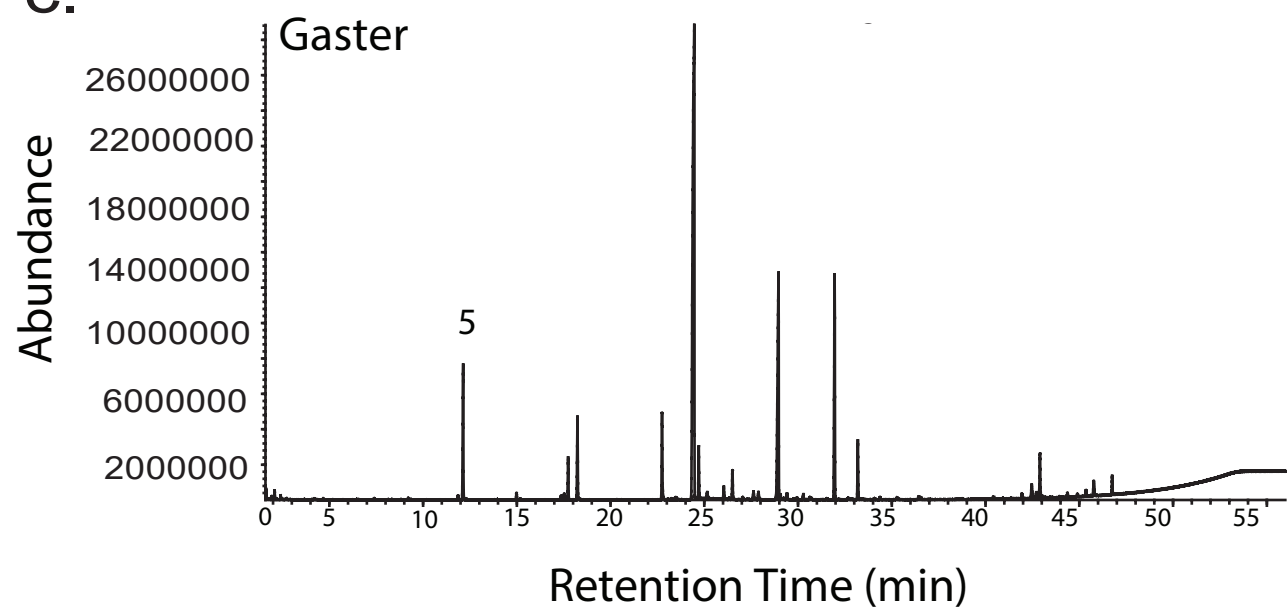

Supplement: Supplementary file 10 — Supplementary file10 (PDF 827 KB) Figure S4. Chemical compounds in the ant body. Gas-chromatographic representation of one sample of 5 pooled workers (a), 5 mesosomas (b) and 5 gasters (c). Compounds found in the head are numbered and can be found in Table 2 [file 10886_2023_1407_MOESM10_ESM.pdf]

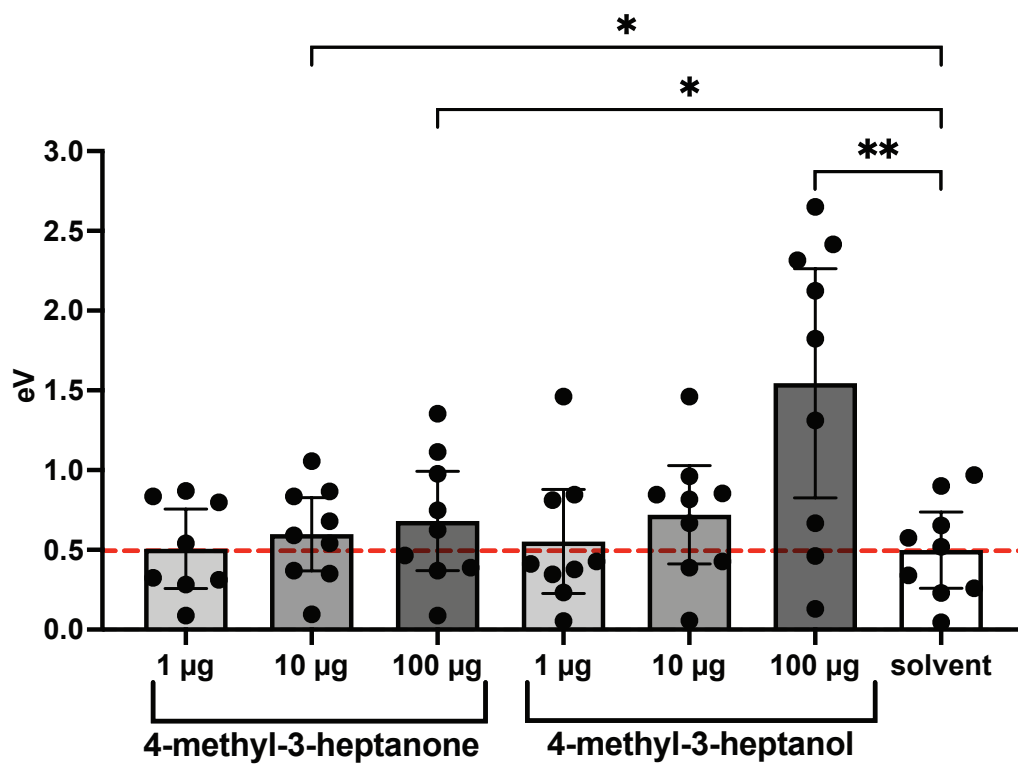

Supplement: Supplementary file 11 — Supplementary file11 (PDF 393 KB) Figure S5. Antennal detection of candidate alarm pheromone components. Results from EAG recordings in response to 1 μg, 10 μg, and 100 μg of 4-methyl-3-heptanone or 4-methyl-3-heptanol and the solvent control pentane. In total, 9 antennae were tested, except for the 1 μg 4-methyl-3-heptanone condition where 8 antennae were tested. Statistical comparisons were made using a mixed-effects analysis with a Geisser-Greenhouse correction and Dunnett’s multiple comparisons test to compare the response to each compound with the solvent control. *p<0.05, **p<0.01, ***p<0.001, ****p<0.0001 [file 10886_2023_1407_MOESM11_ESM.pdf]

**a.**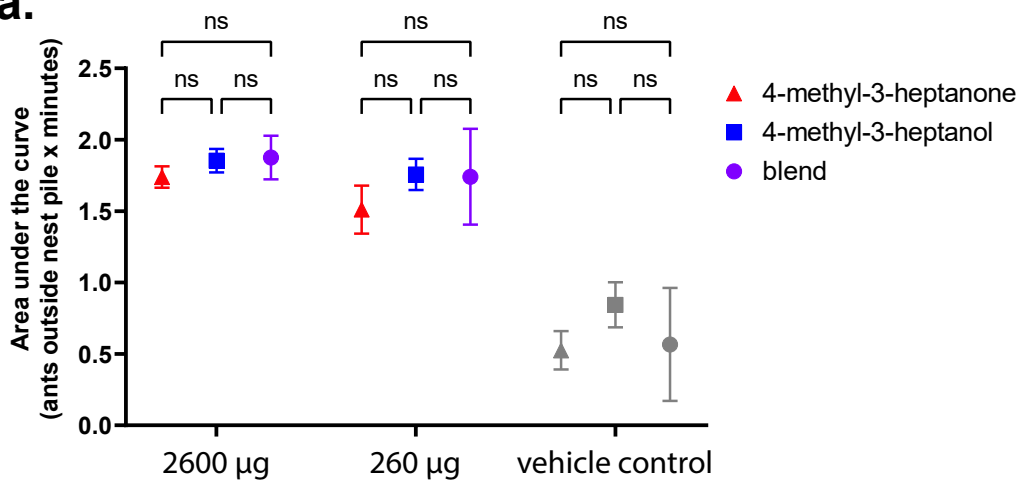**b.**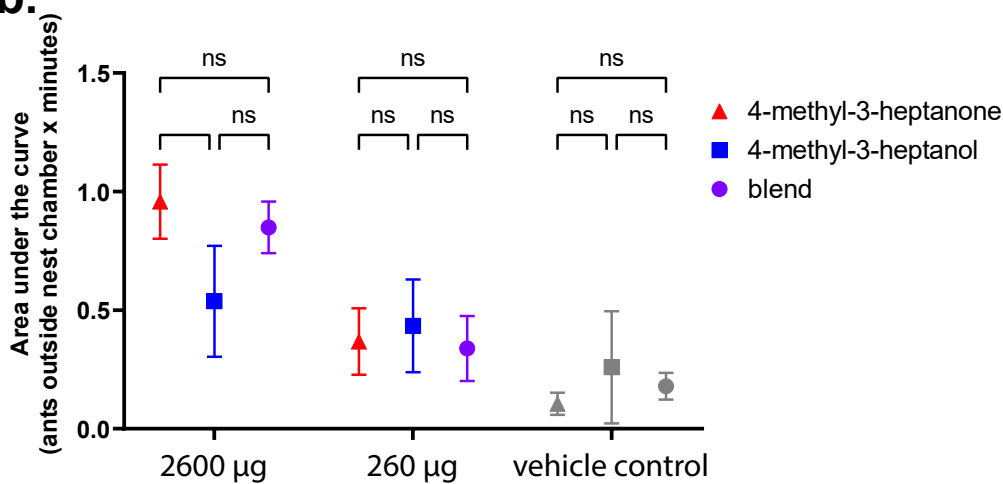**c.**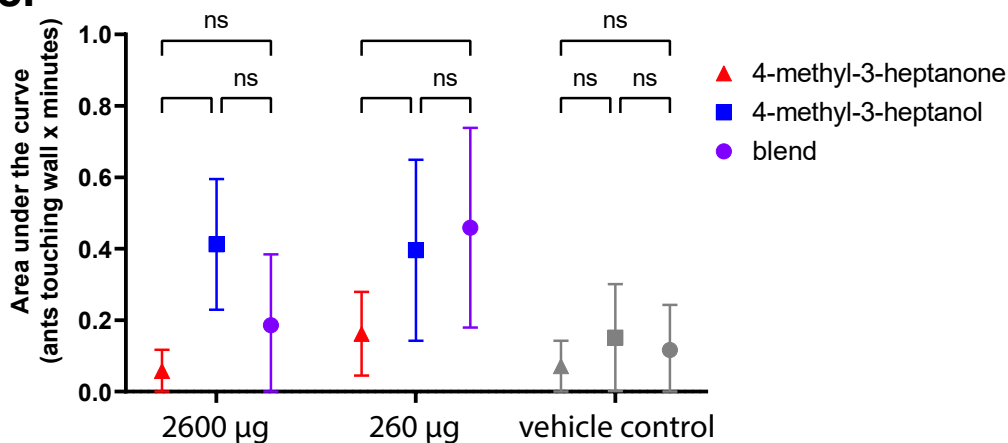

Supplement: Supplementary file 12 — Supplementary file12 (PDF 192 KB) Figure S6. Comparison of behavioral responses to candidate alarm pheromone components and the synthetic alarm pheromone blend. Area under the curve the first 2 minutes after adding the stimulus for ants outside the nest pile (a), ants repelled from the compound(s) (b), and ants attracted to the compound(s) (c). The two compounds and blend were tested in a separate set of experiments and a vehicle control (in grey) was run for each set of experiments. Each datapoint indicates the mean, and error bars represent the 95% confidence intervals. Statistical comparisons were performed using a 2-way ANOVA with Tukey’s multiple comparisons tests to compare the different compounds and blend across concentrations. *p<0.05, **p<0.01, ***p<0.001, ****p<0.0001 [file 10886_2023_1407_MOESM12_ESM.pdf]
